# Ion Depletion Microenvironments Mapped at Active Electrochemical Interfaces with *Operando* Freezing Cryo-Electron Microscopy

**DOI:** 10.1021/acsenergylett.4c00622

**Published:** 2024-05-01

**Authors:** Nikita S. Dutta, Peter J. Weddle, Oscar Hathaway, Mowafak Al-Jassim, Katherine Jungjohann

**Affiliations:** † Materials, Chemical, and Computational Science Directorate, National Renewable Energy Laboratory, Golden, Colorado 80401, United States; ‡ Mechanical and Thermal Engineering Sciences Directorate, National Renewable Energy Laboratory, Golden, Colorado 80401, United States

## Abstract

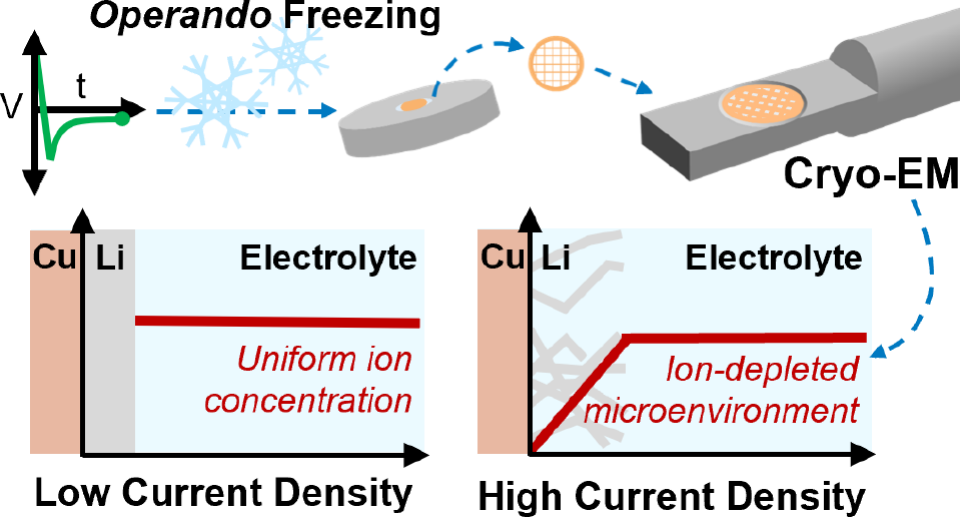

Interfacial structural and chemical evolution underpins safety, energy density, and lifetime in batteries and other electrochemical systems. During lithium electrodeposition, local nonequilibrium conditions can arise that promote heterogeneous lithium morphologies but are challenging to directly study, particularly at the nanoscale. Here we map chemical microenvironments at the active copper/electrolyte interface during lithium electrodeposition, presenting *operando* freezing cryogenic electron microscopy (cryo-EM), a new method, to lock in structures arising in coin cells. We find local ion depletion is correlated with lithium whiskers but not planar lithium, and we hypothesize that depletion stems from root-growing whiskers consuming ions at the growth interface while also restricting ion transport through local electrolyte. This can allow dangerous lithium morphologies to propagate, even in concentrated electrolytes, as ion depletion favors dendritic growth. *Operando* freezing cryo-EM thus reveals local microenvironments at active electrochemical interfaces to enable direct investigation of site-specific, nonequilibrium conditions that arise during operation of energy devices.

Achieving high-energy-density batteries with high-rate capabilities and long lifetimes is critical to electrifying transportation and meeting decarbonization goals. Lithium metal batteries offer promising energy densities but continue to be hampered by poor lifetimes and safety concerns.^1^ Local structural and chemical evolution at active electrochemical interfaces is fundamental to these issues; for instance, during Li plating, the Li-ion concentration adjacent to the active surface can become depleted, leading to a concentration gradient from the surface into the bulk electrolyte over a region known as the diffusion layer.^2,3^ Depletion is a key factor in dendritic Li deposition, as locally higher Li^+^ concentrations at the tips of Li protrusions compared to the surface promotes unstable, directional growth,^3^ which, in turn, can lead to short circuits, capacity loss, and catastrophic battery failure.^4^ Yet, while ion depletion is only expected to occur under certain conditions, such as high current densities or low salt concentrations,^2^ dendrites have been observed outside of these parameters.^5−8^ Moreover, they initiate orders of magnitude more quickly than is predicted by classical models of ion depletion in many common electrolytes.^9^ These discrepancies raise questions regarding the nature of kinetic limitations that arise during Li electrodeposition and eventually lead to failure.

One explanation for disagreement between classical models and observations is the role of local nonequilibrium environments. Dendrite growth is typically observed as a sudden transition after other Li morphologies, such as whiskers or moss, with their accompanied solid electrolyte interphase (SEI) layers have already formed.^7,10^ While these initial morphologies appear less problematic than dendrites, as they are unable to penetrate the separator and short the cell,^8^ they can create locally heterogeneous environments during Li deposition that further promote nonuniform, and potentially dendritic, growth. For instance, high-aspect-ratio growth protrusions such as Li whiskers are thought to induce locally enhanced electric fields along their lengths, influencing local ion distributions and current densities,^11^ while mechanical properties and surface diffusion behavior of the SEI adds further heterogeneity by favoring or disfavoring Li insertion at certain locations.^7,11,12^ These types of effects lead to micrometer-scale regions with unique local environments, wherein dendrites can nucleate even when not predicted by classical models considering equilibrium conditions across the cell. Thus, understanding Li morphological evolution in practical batteries and reconciling experimental observations with theories of electrodeposition require uncovering the role of local microenvironments in inducing regions of unstable growth that go on to dominate failure behavior.

Unfortunately, local structures and chemistry at active electrochemical interfaces are challenging to study directly, particularly at the nanoscale. *In situ* or *operando* optical spectroscopies, such as Fourier-transform infrared (FTIR) or Raman, offer high chemical sensitivity,^13,14^ and *operando* X-ray microscopy can nondestructively map morphological evolution in practical batteries;^15,16^ however, neither of these can achieve combined structural and chemical mapping at the nano- or atomic scale. Electron microscopy is the ideal candidate to complement these techniques at higher spatial resolutions, but while *in situ* electron microscopy can provide insight into model electrochemical systems,^17,18^ it cannot replicate the form factors commonly used in battery research, nor the particular conditions associated with them (e.g., stack pressure, electrolyte volume, etc.). Moreover, reaching the high spatial resolutions theoretically afforded by electron microscopy is complicated in *in situ* techniques by the sensitivity of liquid electrolytes, lithium, and other battery materials to electron beam irradiation. Cryogenic electron microscopy (cryo-EM) has emerged as a solution to the latter problem, enabling multimodal studies of beam-sensitive electrode or electrolyte materials and their interfaces;^19−22^ however, traditional cryo-EM sample preparations for battery materials are *ex situ* and time-consuming. Batteries must be disassembled under an inert atmosphere after electrochemical cycling, a process that takes on the order of 10^3^ s and often damages or destroys solid–liquid interfaces by allowing the electrolyte to dry before freezing.^19,21,23^ Even when care is taken to preserve and vitrify a layer of electrolyte for cryo-EM,^20,24^ ion diffusion coefficients are in the range of 10^–6^ cm^2^ s^–1^ in typical battery liquid electrolytes^25^ and diffusion layers are expected to be 10^–6^–10^–5^ m thick;^26,27^ thus, *operando* ion concentration profiles should relax well before *ex situ* freezing occurs. This makes it impossible to directly visualize the local microenvironments and resulting kinetic limitations that underpin safety and stability in practical batteries by using existing characterization techniques.

Here, we address this challenge by developing a method for *operando* freezing of active electrochemical interfaces and leverage it to study ion depletion during Li electrodeposition in coin cells. Using a custom *operando* electrochemical plunge freezer and modified coin cell designed to allow cold removal of active material, we are able to flash freeze samples at different time points during Li electrodeposition onto copper and use cryogenic scanning transmission electron microscopy (cryo-STEM) energy dispersive X-ray spectroscopy (EDS) to map elemental composition profiles across the active Cu/electrolyte interface. We observe local ion depletion in systems with a whisker-like Li morphology, in contrast to systems with a planar Li morphology, where directional versus planar Li growth is correlated with higher or lower current density (10 or 0.5 mA cm^–2^), respectively, as in the literature.^28^ We find that ion depletion is not predicted for either current density based on a finite-element model of planar Li deposition; thus, we hypothesize that the observed depletion is directly related to the whisker-like morphology and arises due to a combination of fast Li consumption during whisker growth and locally restricted ion transport through the electrolyte surrounding the whiskers. These results provide a mechanistic explanation for why ion-depleted microenvironments can occur even in concentrated electrolytes and demonstrate how cryo-EM with *operando* freezing can preserve electrochemically active solid–liquid interfaces to enable a better understanding of the local structural and chemical evolution that underlies the performance of batteries and other energy devices.

To capture the current-collector/electrolyte interface during Li deposition in its active electrochemical state, we develop a method for *operando* freezing of battery samples consisting of (1) a plunge freezer with integrated electrochemical control to flash freeze samples while current or bias is applied and (2) a modified coin cell with a copper transmission electron microscopy (TEM) grid current collector and retrieval window to facilitate removal of the electrodeposited Li for cryo-EM characterization after freezing. This technique, illustrated in Figure 1 and detailed in Experimental Methods in the Supporting Information, enables temporal control of the point of plunge freezing within an electrochemical experiment with 0.09 s precision, reducing the time delay in cryo-EM sample preparation for batteries by ∼4 orders of magnitude over traditional methods and ensuring that materials of interest are frozen in their native device environment while current or bias is continuously applied. The Cu TEM grid with electrodeposited Li is then retrieved for cryo-EM characterization through a window in the modified coin cell case, while remaining submerged in cryogen (Figures S1 and S2); thus, the sample remains cold and protected from air exposure throughout preparation.

**Figure 1:**
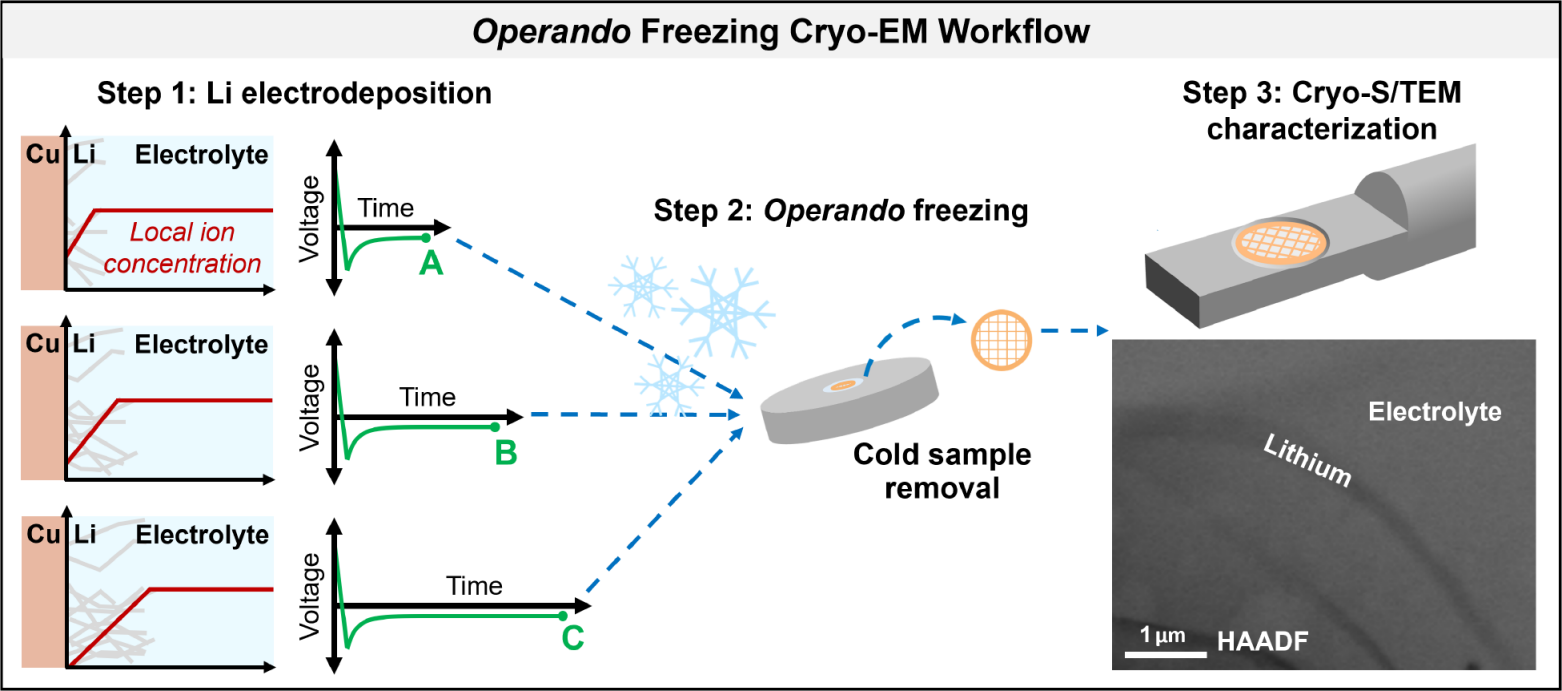
*Operando* freezing cryo-EM workflow. Step 1: Li is electrodeposited onto Cu TEM grid current collectors in modified coin cells. Step 2: Cells are *operando* plunge-frozen at different time points of interest. The grid is then retrieved through a window in the modified cell, while remaining submerged in cryogen. Step 3: Grid is transferred for cryo-S/TEM analysis. A vitrified layer of electrolyte is retained on the sample to enable study of solid–liquid interfaces, frozen in an active electrochemical state.

Figure 1 shows whisker-like Li growth within a matrix of frozen electrolyte after *operando* freezing during Li electrodeposition onto Cu. Here, Z-contrast in the cryo-STEM high-angle annular dark-field (HAADF) image makes the Li appear darker than the surrounding electrolyte due to its lower atomic number.^24^ Cryo-STEM EDS of an area of bulk carbonate electrolyte confirms the expected composition, importantly without notable oxygen or nitrogen contamination from the air or cryogen (Figure S3). The electrolyte layer is confirmed to be amorphous by cryo-TEM selected area electron diffraction (SAED) (Figure S3)—a standard check in cryo-EM to verify that samples have been cooled quickly enough to avoid structural artifacts from crystallization. Note that measurements of the effective bulk freezing rate show it takes several seconds for ion transport across the entire coin cell to be kinetically halted (Figure S3); however, the lack of crystallinity in cryo-SAED analyses confirms that *operando* plunge freezing is sufficient to vitrify the thin layer of electrolyte attached to the TEM grid current collector.

We present *operando* freezing to preserve and study ion concentration gradients at the active current-collector/electrolyte interface during Li electrodeposition. Modified coin cells consisting of Cu TEM grids versus Li metal are frozen at varying time points in Li deposition at 0.5, 2.5, or 10 mA cm^–2^. We then take cryo-STEM EDS line scans from the edge of a Cu grid bar into the electrolyte that fills the pore in the Cu grid to investigate chemical composition gradients at the current-collector/electrolyte interface (as shown in Figure 2). While composition gradients in the intact cell should extend three dimensionally around each grid bar, the sample extracted for cryo-STEM is planar, and thus, we are mapping compositions laterally across the current collector plane. Note that as the thickness of the frozen electrolyte layer is determined by how the TEM grid peels off the electrode stack during cold removal from the modified coin cell, it is not well controlled and is often too thick to clearly visualize Li growth in the electrolyte. Thickness control can be addressed in future development of the *operando* freezing method; however, the current method still yields areas suitable for EDS analysis.

**Figure 2:**
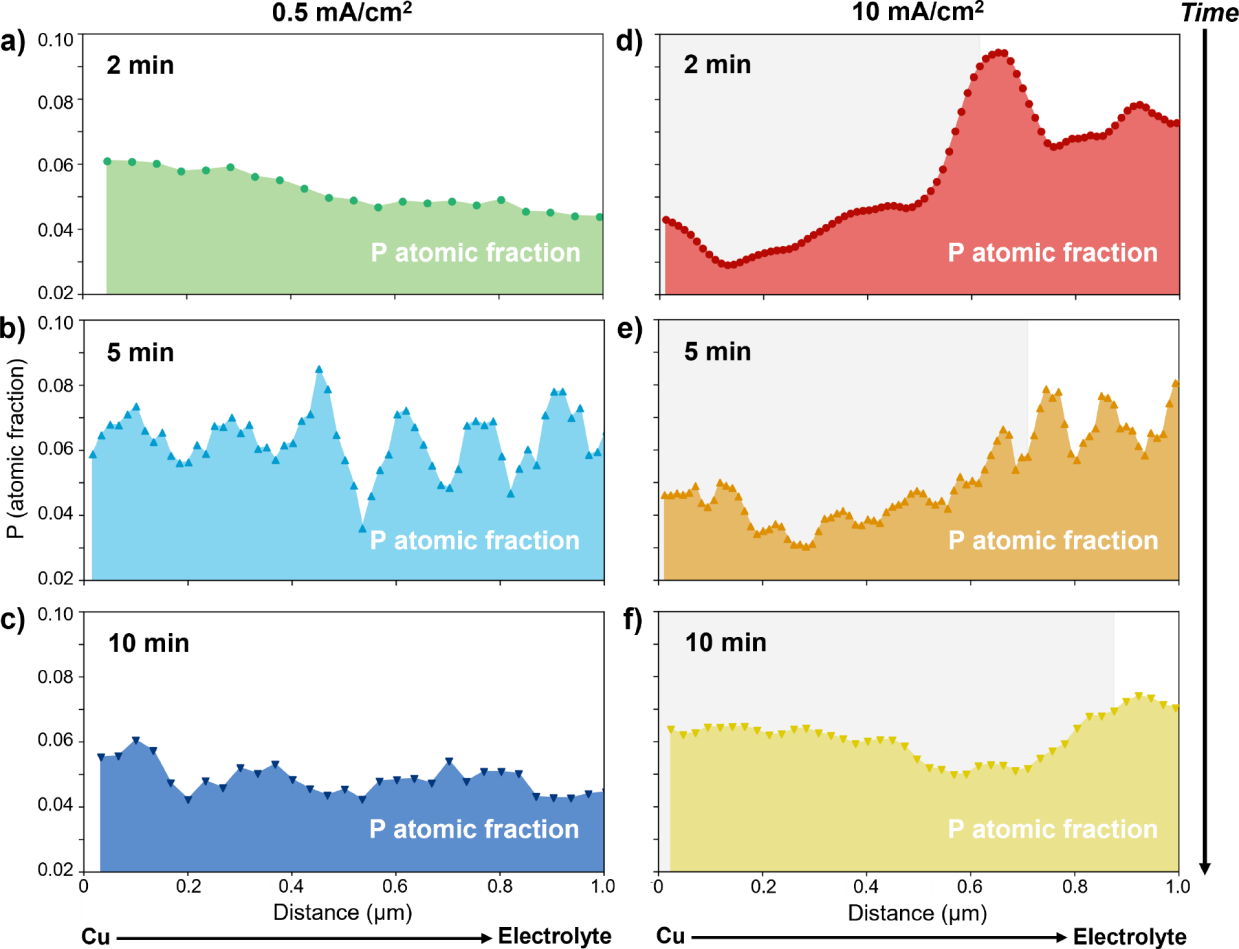
Composition at the Cu/electrolyte interface. Cryo-STEM EDS phosphorus atomic fraction profiles scanning from the Cu current collector to the electrolyte for samples *operando* frozen at 2, 5, or 10 min of Li electrodeposition at 0.5 mA cm^–2^ (a–c) or 10 mA cm^–2^ (d–f). Low-current-density samples show a relatively constant composition with increasing distance from the Cu surface, while high-current-density samples show an initial P gradient (gray shaded region) that plateaus.

Figure 2 shows the phosphorus atomic fractions from example EDS line scans at varying time points for the lowest and highest current densities; example STEM images and corresponding EDS maps are given in Figure S4. Mapping the P atomic fraction serves as an approximate proxy for the distribution of PF_6_^–^ ions within the electrolyte, which follows the Li^+^ distribution due to local electroneutrality.^29^ Here, P is chosen over F because the clear separation of its Kα peak from other electrolyte component peaks aids quantification at the low electron doses needed for cryo-EM (Figure S3), and the P atomic fraction is calculated considering only EDS-detectable electrolyte components C, O, P, and F. The Cu signal is used to calibrate the *x* = 0 position to the edge of the Cu grid bar across all samples (Figure S5) but is then removed from the atomic fraction calculation and the fraction of the remaining elements is renormalized. While the measured composition of these elements must combine that of the bulk electrolyte with that of the SEI on the deposited Li, the latter is only expected to be 10–20 nm thick,^24^ so when scanning across micron length scales, the signal should be dominated by the composition of the bulk electrolyte.

Local chemical compositions and composition profiles show heterogeneity across the samples. This points to the existence of heterogeneous local environments across the cell but likely also relates to the fact that (1) EDS provides a semiquantitative view of the samples, particularly due to low signal-to-noise ratios when using low electron doses, that highlights spatial variations in composition with better accuracy than the absolute composition and (2) cryo-EM characterization is destructive, so each combination of time point and current density represents a unique coin cell and thus introduces potential for batch-to-batch variation. In general, we observe a constant composition extending from the Cu surface into the electrolyte at low current density (0.5 mA cm^–2^; Figure 2a–c), while at high current density, we frequently observe an initial composition gradient that later plateaus (10 mA cm^–2^; Figure 2d–f). Figure S6 gives statistics on how often a composition gradient is observed across all EDS line scans—more than half the time for samples deposited at 10 mA cm^–2^ and very rarely (2 out of 13 scans) for samples deposited at 0.5 mA cm^–2^. Samples deposited at the intermediate current density (2.5 mA cm^–2^) also frequently show a composition gradient, in contrast to samples deposited at 0.5 mA cm^–2^ even when deposited to the same capacity (Figure S7). Moreover, samples frozen without any applied current do not show a composition gradient (Figure S8), suggesting gradient formation is related to the active electrochemical state of the interface and, more specifically, to the current density.

The presence or absence of a P gradient at the Cu/electrolyte interface is also correlated to the Li deposition morphology. Figure 3 shows *ex situ* scanning electron microscopy (SEM) images of Li deposited onto Cu grids at 0.5 or 10 mA cm^–2^, either for the same amount of time (10 min; Figure 3a,c) or to the same capacity (1.7 mAh cm^–2^; Figure 3b,c). At the lower current density, Li covers the Cu grid bars with dense, uniform grains about 1 μm in size after either 10 or 200 min of deposition, though some Li whiskers or moss are apparent at the longer time. The pores of the Cu TEM grid and edges of the Cu bars remain clear, without any appreciable Li growth into the pore (Figure S9). Meanwhile, at the higher current density, the Li shows a whisker-like morphology (Figure 3c) with extensive growth into the pore (Figure S9). This is also observed for the intermediate current density (Figures S9 and S10); thus, a uniform versus whisker-like Li morphology is correlated with current density and the absence or presence of a composition gradient at the Cu/electrolyte interface in cryo-STEM EDS.

**Figure 3:**
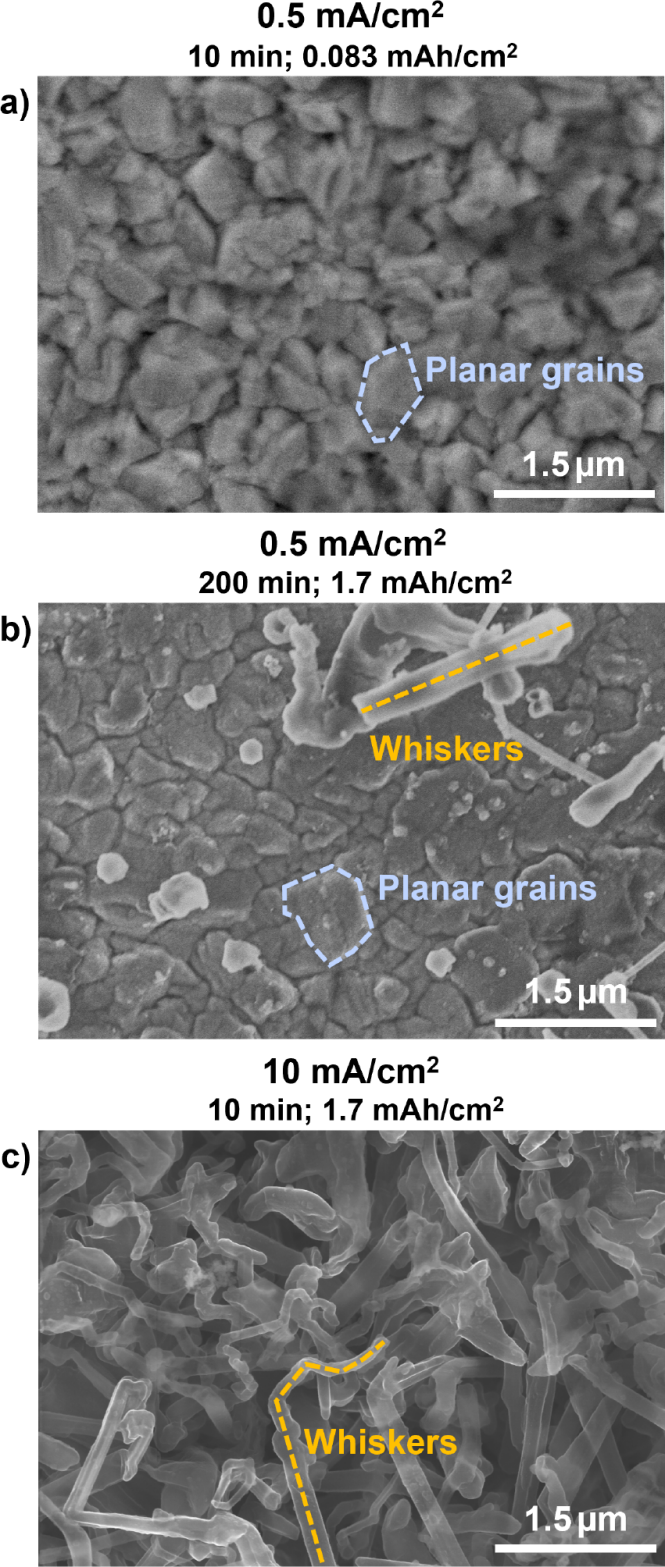
Lithium deposition morphology. SEM images of Li morphology on Cu grids after (a) 10 min of deposition at 0.5 mA cm^–2^ (0.083 mAh cm^–2^), (b) 200 min of deposition at 0.5 mA cm^–2^ (1.7 mAh cm^–2^), and (c) 10 min of deposition at 10 mA cm^–2^ (1.7 mAh cm^–2^). Despite the appearance of some Li whiskers and mossy Li after 1.7 mAh cm^–2^ at 0.5 mA cm^–2^ (b), the overall Li morphology remains largely planar and uniform, unlike that in the 10 mA cm^–2^ case, where dense Li whiskers grow.

Since Li whiskers grow into the open pore of the Cu grid (the direction of the EDS line scans), while uniform Li does not, we hypothesize that the composition gradients observed in the EDS data at high current densities occur within the whisker growth region and result from the dense aggregation of whiskers acting to locally restrict ion transport to the current collector, while Li^+^ ions are quickly consumed at that interface. Previous research has argued that columnar Li whiskers form when stress builds up on the surface of nuclei, in part due to SEI formation, and promotes Li insertion at the current-collector/Li interface that then pushes up the SEI-rich tip.^7,11,12,30−32^ This root growth mechanism places whiskers in contrast to fractal dendrites, which grow from their tip due to the aforementioned mass transport limitations. Thus, even though ion depletion is not thought to be required to *initiate* Li whisker growth, a combination of continuous Li-ion consumption at the Cu/Li interface with restricted ion transport through the Li whisker region may cause ion depletion to occur locally in the electrolyte adjacent to the Cu surface *during* whisker growth, resulting in nonequilibrium microenvironments with the chemical composition gradients observed in cryo-STEM EDS.

To confirm this hypothesis, we employ a finite-element simulation of planar Li electrodeposition at 0.5 and 10 mA cm^–2^, with a model geometry reflective of the modified coin cell, shown in Figure S11. Figure 4 shows the predicted P atomic fraction profile at the Cu/electrolyte interface—starting from the Cu grid bar (*x* = 0) and extending into the electrolyte that fills the pore in the Cu grid, as in cryo-STEM EDS measurements—compared to the profiles observed in cryo-STEM EDS at 2, 5, and 10 min of deposition at each current density. The profiles are normalized by the P atomic fraction 1 μm into the electrolyte to compare the extent of ion depletion near the surface relative to the “plateau” value far from the interface for each sample.

**Figure 4:**
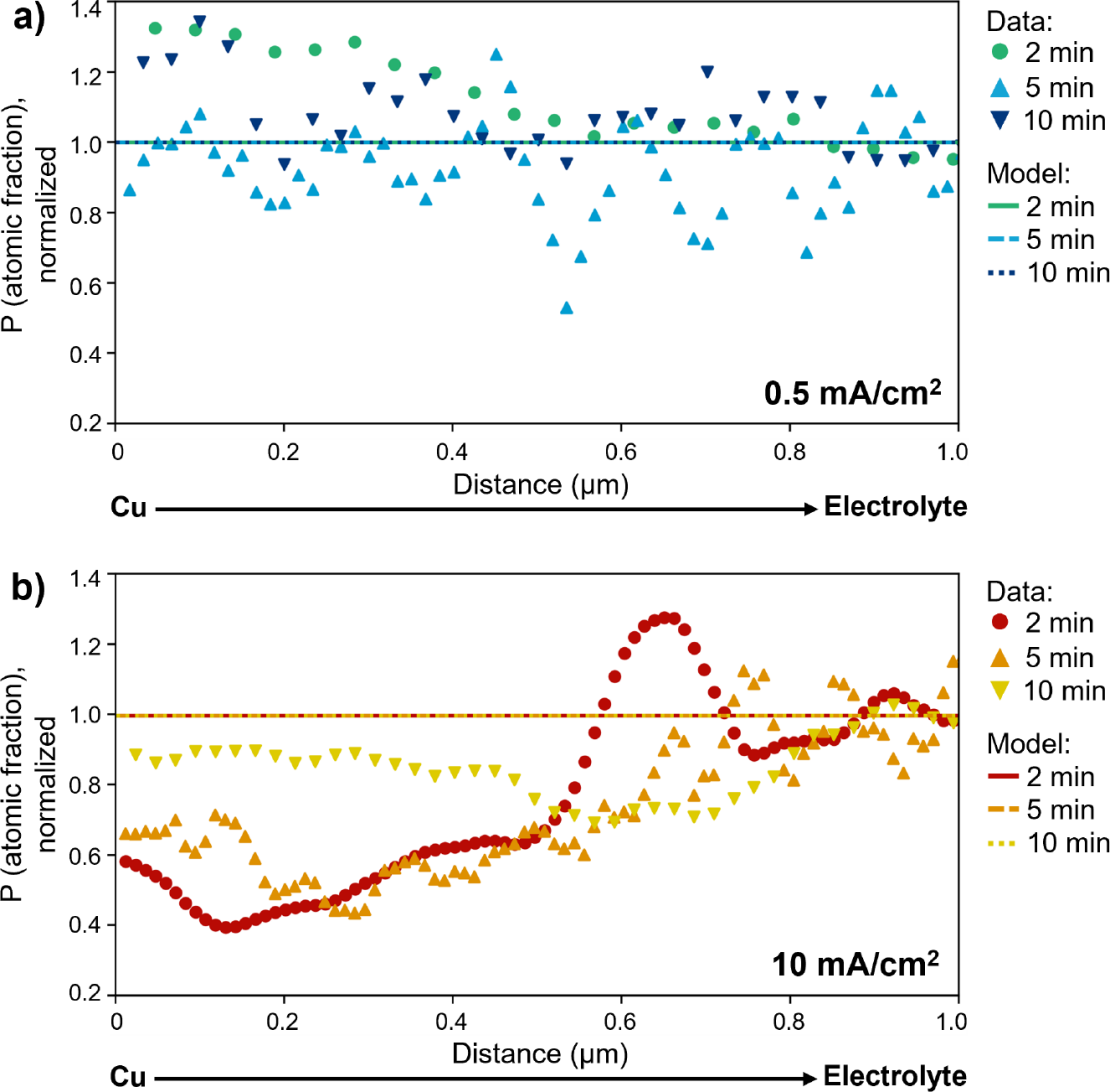
Modeled salt concentration. P atomic fraction at the Cu/electrolyte interface, normalized by atomic fraction 1 μm into electrolyte, predicted by finite-element model (lines) or observed experimentally by cryo-STEM EDS (markers) after 2, 5, or 10 min of Li deposition onto Cu at 0.5 mA cm^–2^ (a) or 10 mA cm^–2^ (b). Virtually no change in P atomic fraction is expected for either current density under the assumption of planar Li deposition, and thus lines representing the modeled profiles at 2, 5, and 10 min overlap.

It is apparent that under the assumption of planar Li growth, negligible ion depletion at the interface is predicted, leading to relatively flat P profiles for both current densities at all three time points (further results on modeled concentrations are available in Figures S12 and S13). This is concordant with results in the literature for the high salt concentrations used in practical battery electrolytes such as Gen2,^33^ as well as with the constant P composition observed at the Cu/electrolyte interface in samples electrodeposited at 0.5 mA cm^–2^, where planar Li growth is observed. For samples deposited at the higher current density, the model suggests that where ion transport through the electrolyte is unrestricted, as in the electrolyte adjacent to a planar Li surface, negligible ion depletion should occur, consistent with the P plateau observed some distance from the Cu surface in cryo-STEM EDS. This indicates that the depleted region is not the traditional “diffusion layer” that would form adjacent to planar Li growth at high enough current densities to induce diffusion limitations, as those are not predicted for the system. Close to the Cu surface, however, the presence of whisker-like Li growth in the pore of the Cu grid invalidates the assumption of planar Li growth and leads to a discrepancy between the minimal ion depletion predicted by the model and the notable depletion observed experimentally. The width of this depleted region grows with time at a rate of approximately 5.0 × 10^–10^ m s^–1^, as shown in Figure 5a. This suggests that the width of the depleted area is related to the increasing length of the deposited whiskers with time, supporting that ion depletion occurs in regions where Li whiskers have grown into the pore in the Cu grid.

**Figure 5:**
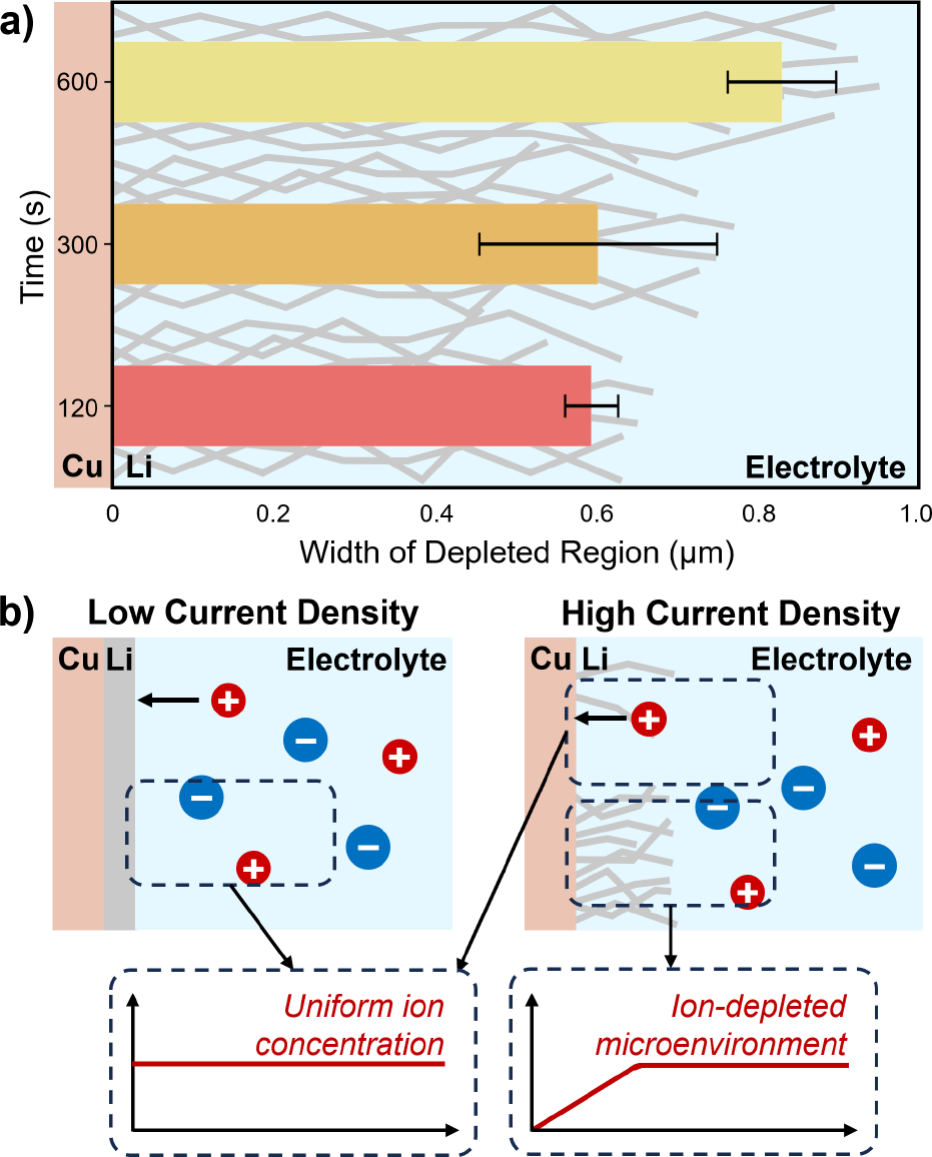
Depleted microenvironment formation. (a) Width of the ion depletion region observed for samples deposited at 10 mA cm^–2^ increases with time. Bars represent an average width from two regions of the sample, with error bars showing their standard deviation to illustrate the scale of width heterogeneity across single samples. (b) Proposed mechanism for formation of depleted microenvironments. At low current densities, Li deposits uniformly and no significant ion depletion occurs in the electrolyte. At high current densities, heterogeneous growth of Li whiskers restricts local ion transport to the current collector, where Li^+^ is rapidly consumed, leading to formation of ion depletion microenvironments.

These results suggest that heterogeneous Li whisker growth can restrict ion transport through the surrounding electrolyte and lead to local ion depletion at the current-collector/electrolyte interface, even for current densities and electrolyte formulations where depletion is not predicted based on diffusion models for planar Li growth, as illustrated in Figure 5b. This points to the importance of the initial Li deposition morphology in potentially initiating a self-propagating cascade of undesirable structural evolution, as the formation of an ion concentration gradient due to Li whisker growth can eventually spur tip growth of fractal dendrites. This transition from blunt, root-growing whiskers to tip-growing fractal dendrites, which can penetrate the separator and short the cell,^8^ has been observed in the literature,^7,34^ but without a mechanistic understanding as to what causes this transition. These results offer an explanation that the transition is mediated by regions of local ion depletion at the current-collector/electrolyte interface that arise even in concentrated electrolytes, due to restricted transport through the rapidly growing Li whiskers, which eventually make root growth unfavorable and push the system toward dendritic tip growth. This insight bridges a critical gap between a theoretical understanding of kinetic limitations in lithium metal batteries and those that arise locally in practical systems and go on to dominate battery failure.

In summary, we have presented, for the first time, a method for *operando* freezing of samples during Li electrodeposition onto Cu in coin cells that preserves the active electrochemical interface in a vitrified state suitable for cryo-EM imaging and spectroscopy. We show that local ion depletion at the current-collector/electrolyte interface occurs in samples with a whisker-like Li morphology, in contrast to samples with a planar Li morphology, and we hypothesize that this stems from the fact that root-growing whiskers quickly consume Li^+^ at the Cu/Li interface, while also forming a dense mass that locally restricts transport of ions to that interface. Importantly, these results could not have be achieved using traditional cryo-EM techniques, which require removing materials from their native device environment and stimulation before freezing occurs. Rather, *operando* freezing preserves solid–liquid interfaces in their active electrochemical states to enable the direct exploration of local microenvironments and kinetic limitations that arise during device operation. This method has an impact beyond batteries as a means to explore *operando* structural and chemical evolution at other electrochemical interfaces that are similarly critical to energy systems yet challenging to characterize at the nanoscale, such as those involved in fuel cells, electroplating, or electrocatalytic fuel production. Thus, these results stand to compound the impact of cryo-EM in materials science, revealing the challenging-to-characterize local environments that are central to the performance of electrochemical energy systems.

## Experimental Methods

Full details on the experimental methods, including modified coin cell preparation, *operando* freezing, and electron microscopy characterization and analysis, as well as on the finite-element model (with further model results depicted in Figures S12 and S13) are provided in the Supporting Information.

## Supporting Information Available

Full experimental methods and finite-element model details, including additional figures pertaining to validation of the method development, experimental results, and analyses (PDF)
