# Supplementary material for: Ion Depletion Microenvironments Mapped at Active Electrochemical Interfaces with Operando Freezing Cryo-Electron Microscopy
Source: ACS Energy Lett. 2024 May 1;9(5):2464–71. doi: 10.1021/acsenergylett.4c00622 (PMC11091875; doi:10.1021/acsenergylett.4c00622)
Supplement: Supplementary file 1 — nz4c00622_si_001.pdf [file nz4c00622_si_001.pdf]

Supporting Information for:

Ion Depletion Microenvironments Mapped at Active Electrochemical Interfaces with *Operando* Freezing Cryo-Electron Microscopy

Nikita S. Dutta[1]*, Peter J. Weddle[2], Oscar Hathaway[1], Mowafak Al-Jassim[1], and Katherine Jungjohann[1]

[1]*Materials, Chemical, and Computational Science Directorate, National Renewable Energy Laboratory, Golden, Colorado 80401, United States*

[2]*Mechanical and Thermal Engineering Sciences Directorate, National Renewable Energy Laboratory, Golden, Colorado 80401, United States*

**E-mail: nikita.dutta@nrel.gov*

Experimental Methods

Modified Coin Cell Preparation

Modified coin-cell cans were prepared by punching a ≈7 mm diameter hole into standard stainless steel CR2032 cans. On the inside of each can, a square with 0.5 in sides cut from a 100 μm thick sapphire wafer was placed over the hole and attached using a thin ring of Devcon 14278 epoxy. Once the epoxy was cured, a circle of 100x100 Cu mesh was cut to the inner diameter of the can, and then cut into two sections with one slightly larger than the other (about 2/3 of the way across the diameter). The two pieces of mesh were lined up on the inside of the can, over the sapphire, and the edge of each piece was welded to the can to create a conductive pathway across the window.

Modified cell assembly was performed in an argon-atmosphere glovebox. A PELCO 300 mesh Cu TEM grid (used as-is) was slid under the Cu mesh such that it rested over the hole in the can. Note that while Li did deposit onto the conductive Cu mesh surface during electrodeposition, this did not prevent Li from depositing onto the Cu TEM grid as well, nor did it cause an issue during removal of the TEM grid after freezing. 75 μL of Gen2 electrolyte (3:7 w/w ethylene carbonate:ethyl methyl carbonate with 1.2 M $LiPF_6$) was deposited over the grid and mesh, followed by a 19 mm diameter Celgard separator, gasket, 5/8 in punch of Li metal foil (with the surface layer scraped off), stainless steel spacer, wave spring, and cap. Finally, all components were pressed for 2 s using an automatic hydraulic crimper and rested for at least two hours before cycling.

Cells were monitored for electrolyte leakage throughout crimping and resting, and any cell that showed leakage (usually associated with a visible crack in the window) was disposed. Modified cells displayed consistent electrochemistry with conventional coin cells comprised of the same materials, without the presence of additional voltage plateaus or other features that would suggest a reaction between the electrolyte and epoxy (Fig. S2). Thus, the modified cell construction was determined to provide a sufficient, inert seal for the electrolyte and voltage range tested. Future work considering other electrolyte formulations or voltage windows should

reconfirm that modified cells show comparable electrochemistry to conventional cells as a standard check for this technique.

Operando Plunge Freezing

Cells were cycled in an *operando* plunge freezer consisting of a CHI 760E electrochemical workstation, modified Thermo Fisher Vitrobot Mark IV, and custom-built *operando* freezing sample holder. Control of the workstation was integrated with control of the plunge freezer via a LabJack data acquisition system and Python. In order to trigger plunge freezing at a particular time point in Li electrodeposition, the actuator speed was measured and calibrated for by recording 10 instances of plunging at 240 fps and taking the average amount of time between when plunging was triggered and when the sample was fully submerged in the cryogen (1.51 s). The precision of triggering plunging based on a calibrated test time was determined by taking the standard deviation of these times (0.09 s). The effective bulk freezing rate was also measured by *operando* freezing a modified Li-Cu cell (prepared as described above) during linear sweep voltammetry from 3 V to 1 V at a rate of 0.1 V s^{-1} and taking the time for the output current to decay to 0, indicating that ions and electrolyte throughout the cell were immobilized. In addition, as the small TEM grid current collector was expected to cool much faster than the bulk cell, the sample freezing rate was validated using cryo-TEM SAED to check that the electrolyte was amorphous after plunge freezing (Fig. S3), as is standard in cryo-EM of liquids.

For Li electrodeposition studies, the environmental chamber of the Vitrobot was set to 25°C and ambient relative humidity (~40%). Current was applied at either 0.5 mA cm^{-2}, 2.5 mA cm^{-2}, or 10 mA cm^{-2}—as the combined surface area of the Cu mesh and TEM grid was larger than that of the Li metal punch, the Li metal surface area was used in calculating current density. Cells were plunge frozen 2, 5, and 10 min into current application (calibrating for speed of the plunge actuator as described above), with current flowing continuously throughout plunging into liquid nitrogen (LN_2). LN_2 was selected as the cryogen due to its non-flammable nature; flammable cryogens such as ethane were determined to be hazardous when plunging electrified samples. After the cell was fully submerged in LN_2, still held by the custom sample holder, the electrochemical workstation was powered off and the cell was released from the sample holder into the cryogen dewar by pushing it out with cooled plastic tweezers. Note that as characterization was destructive, each combination of current density and time consisted of a new coin cell.

Once the cell was loose in the cryogen dewar, the dewar was gently moved from the Vitrobot platform to the lab bench. Using cooled plastic tweezers, the cell was manipulated to sit with the window facing up in the bottom of the dewar. Long tweezers were used to hold the top of a cooled nail and position its tip on the window, just to the side of TEM grid rather than directly over it to avoid damage. A hammer was used to quickly tap the nail and break the window. Cooled fine-tip tweezers were then used to extract the grid and transfer it to a cryo-EM sample box. From here, cryo-transfer to the S/TEM proceeded as normal, closing the box under LN_2 and moving it quickly to the cryo-transfer station of a side-entry cryo-TEM holder or to a cryo-storage dewar or dry shipper for transport to another facility.

Cryo-EM Characterization

Characterization of the Li-Cu half cell without any current application was performed on a Thermo Fisher Scientific Spectra 200 S/TEM, operated at 200 kV, with a Gatan 626 cryo-transfer holder maintained at temperatures below -160°C. All remaining samples were characterized using a Thermo Fisher Scientific Talos F200X G2 S/TEM, operated at 200 kV, with a Gatan Elsa cryo-transfer holder maintained at temperatures below -160°C. Cryo-STEM imaging and EDS was performed at spot size 6 with a 98 mm camera length and semi-convergence angle of 10.50 mrad. EDS maps were acquired using Super-X EDS detection, which leverages four silicon drift detectors positioned symmetrically around the sample to achieve high-efficiency X-ray collection while reducing potential shadowing effects. The EDS dwell time was 2 μs and yielded screen currents around 20 pA.

SEM

Samples for SEM were prepared by assembling coin cells for Li electrodeposition as described above, but using as-is stainless steel coin cell cans (without windows) and pressing for 5 s to seal. No Cu mesh besides the TEM grid was used, as the whole can was already conductive; this did not affect the current density compared to the modified cells as that was still limited by the area of the Li punch. Cells were rested for 2 hrs, after which Li electrodeposition was performed on a Maccor Series 4000 held at 25°C at 0.5, 2.5, or 10 mA cm^{-2} for 10 min. Cells were then removed from the Maccor, brought into an Ar glovebox, and disassembled. The TEM grids were removed from the disassembled cells and gently rinsed in dimethyl carbonate for ~10 min each to remove residual electrolyte. The samples were left to dry under Ar for 2 hrs before mounting onto an SEM stub inside the glovebox using double-sided carbon tape. The stub was then placed in the antechamber of the glovebox and vacuum pumped for an additional 10 min to ensure samples were fully dry. Finally, the stub was brought back into the glovebox, sealed under Ar, and wrapped in Parafilm before removing for transfer to the SEM to minimize air exposure. SEM images were acquired via the secondary electron (SE) detector on a Hitachi S-4800 Scanning Electron Microscope. Images were captured at an accelerating voltage of 3 kV, an emission current of 7 μA, and at working distances between 3.6 and 3.9 mm.

EDS Data Analysis

EDS quantification was conducted in Velox to yield atomic fractions across line scans. Quantification was improved using a pixel averaging pre-filter (typically 5 or 10px) to compensate for low signal-to-noise in EDS acquisitions due to low electron doses. Increasing the number of pixels averaged prior to quantification increased the signal-to-noise and brought the calculated P atomic fraction closer to the theoretical value generated by the finite-element model, at the expense of spatial resolution. However, pixel averaging did not change the qualitative shape of the data (e.g., whether the profile was relatively flat or showed a depleted region); thus, a 5-10px average filter was generally sufficient to improve the quantification while maintaining spatial resolution, but did not change the presence or absence of the depleted region.

After quantification, the P atomic fraction was normalized based on the summed C, P, O, and F atomic fractions, such that the Cu atomic fraction did not influence the final plotted

profiles—this ensured that spatial variation in the plotted P atomic fractions reflected variations in the electrolyte composition, rather than variations in the amount of signal coming from the Cu versus the electrolyte. EDS line scans showing unusual artifacts or contamination (e.g., O-rich features) were excluded from analysis. The remaining scans were calibrated as illustrated in Fig. S5 to align them such that drop-off of the Cu signal (from the edge of the grid bar) occurred at x=0. Calibrated scans were labelled as either showing a relatively flat P composition or an increasing P composition gradient for the statistical analyses shown in Fig. S6. For width measurements of the depleted regions, the two least noisy scans were taken from each of the time points for deposition at 10 mA cm^{-2} and the widths of their depleted regions were averaged.

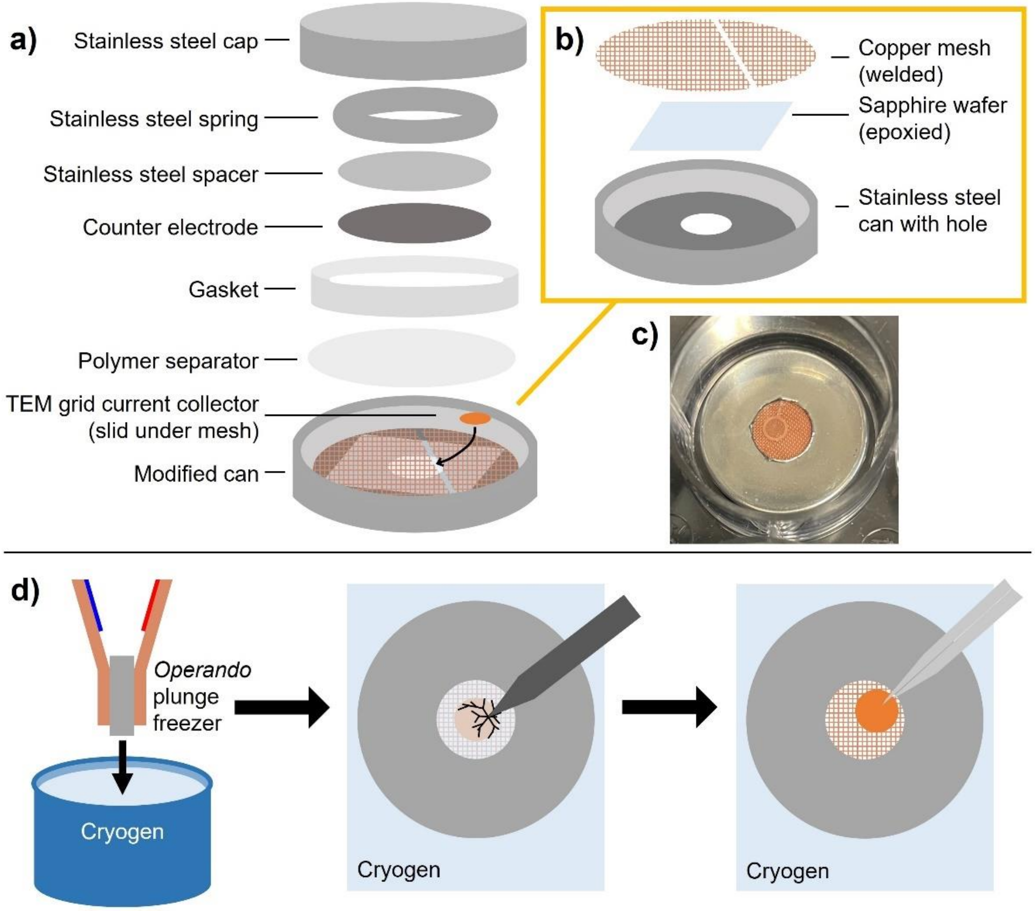

Figure S1: Modified coin cell, *operando* freezing, and cold sample removal process. (a) Diagram of modified coin cell, with close-up of modified can (b) and photo of assembled cell (c). (d) Workflow for cold sample removal: Modified cell is plunge frozen using the *operando* plunge freezer with electrochemical sample holder, the window is broken while submerged under cryogen, and the working electrode is removed for characterization.

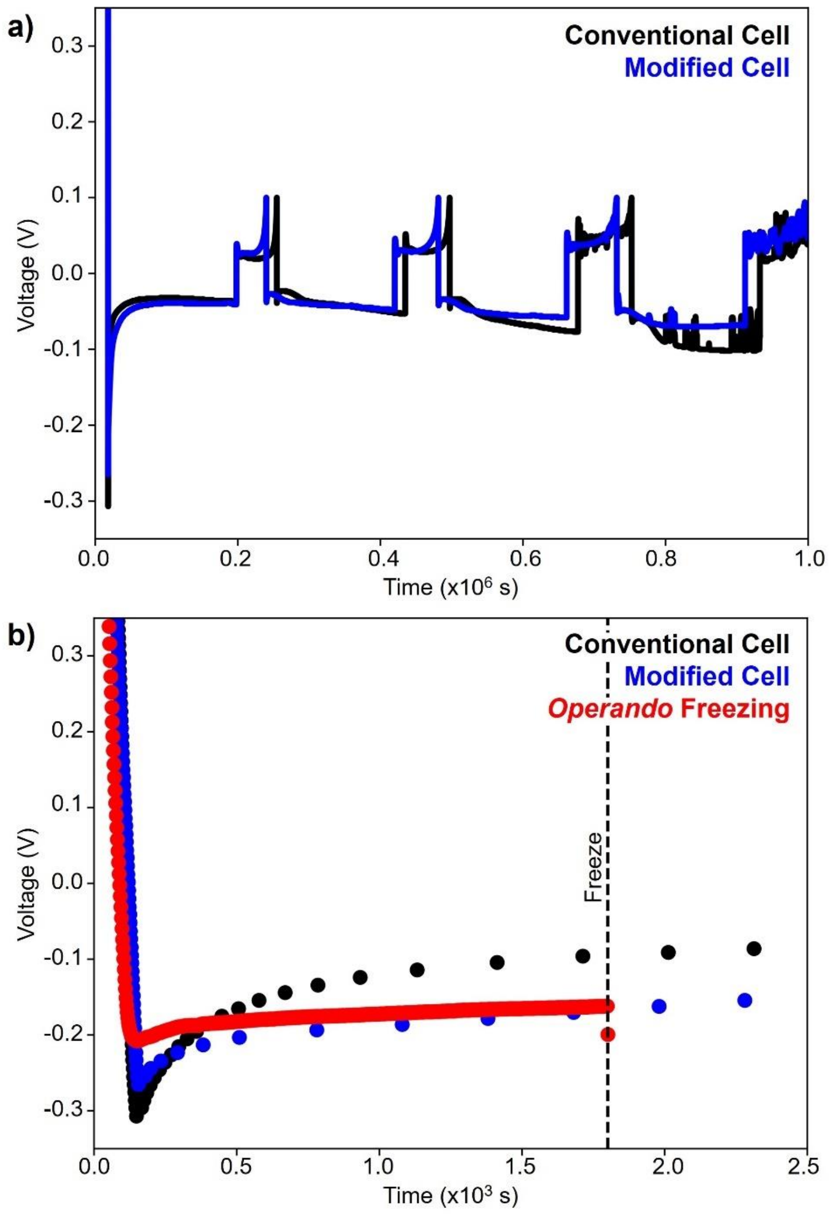

Figure S2: (a) Li electrodeposition onto Cu TEM grids at 0.5 mA/cm^2 in a modified coin cell (blue) shows a similar voltage profile as in a conventional cell (black). (b) Voltage profile during *operando* freezing 30 min into Li electrodeposition at 0.5 mA/cm^2 using modified coin cell and custom electrochemical holder for plunge freezer (red), compared to electrodeposition in conventional (black) or modified (blue) cells using standard Maccor cycler.

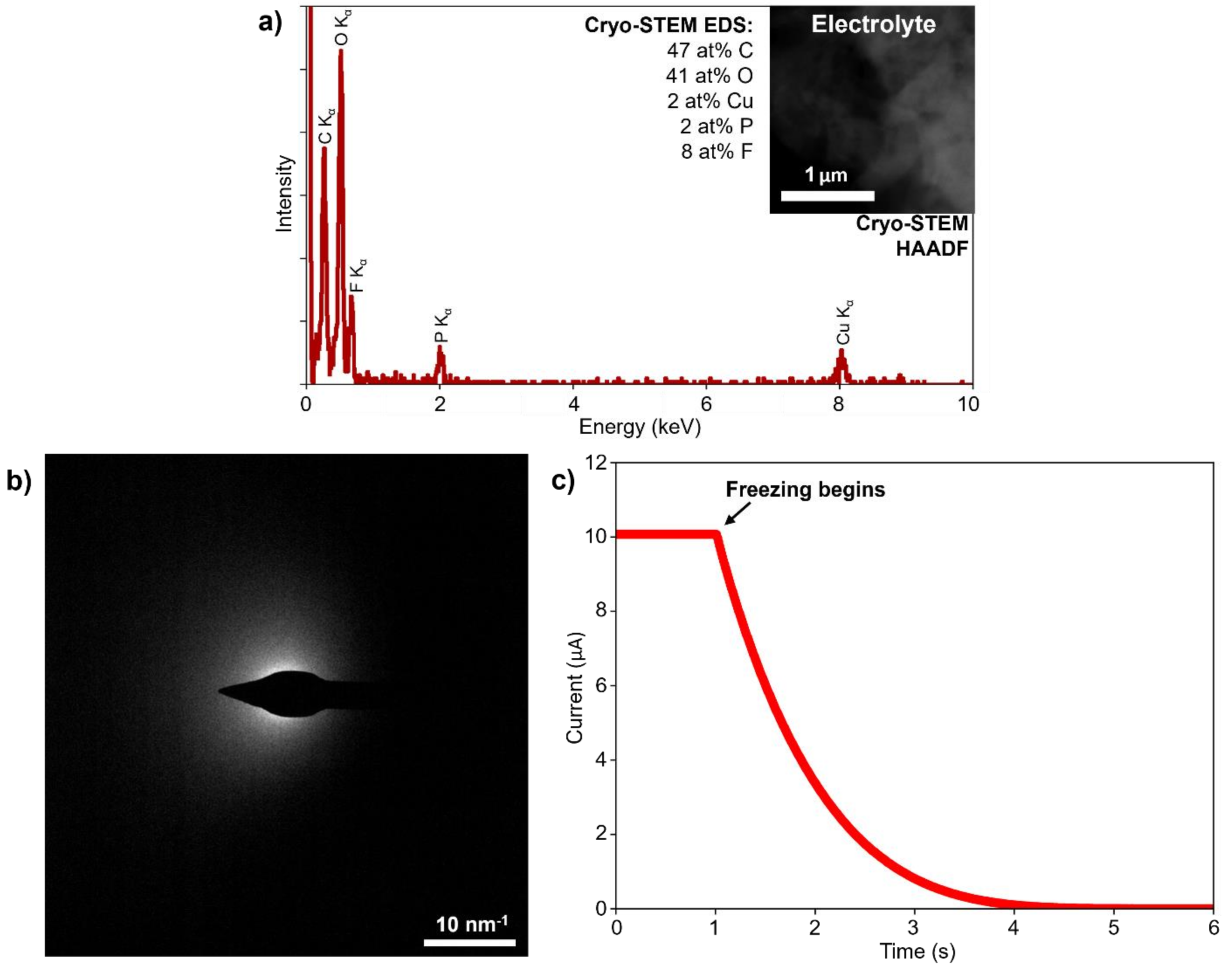

Figure S3: Method validation. (a) Cryo-STEM EDS from region of bulk Gen2 electrolyte shows expected elemental composition, without notable contamination from air or cryogen. (b) Cryo-TEM SAED of sample *operando* frozen during Li deposition onto Cu does not show evidence of electrolyte crystallization during freezing. (b) *Operando* freezing during linear sweep voltammetry of a Li-Cu half cell shows it takes ~3 s for ion transport to be sufficiently kinetically halted such that the output current decays to 0.

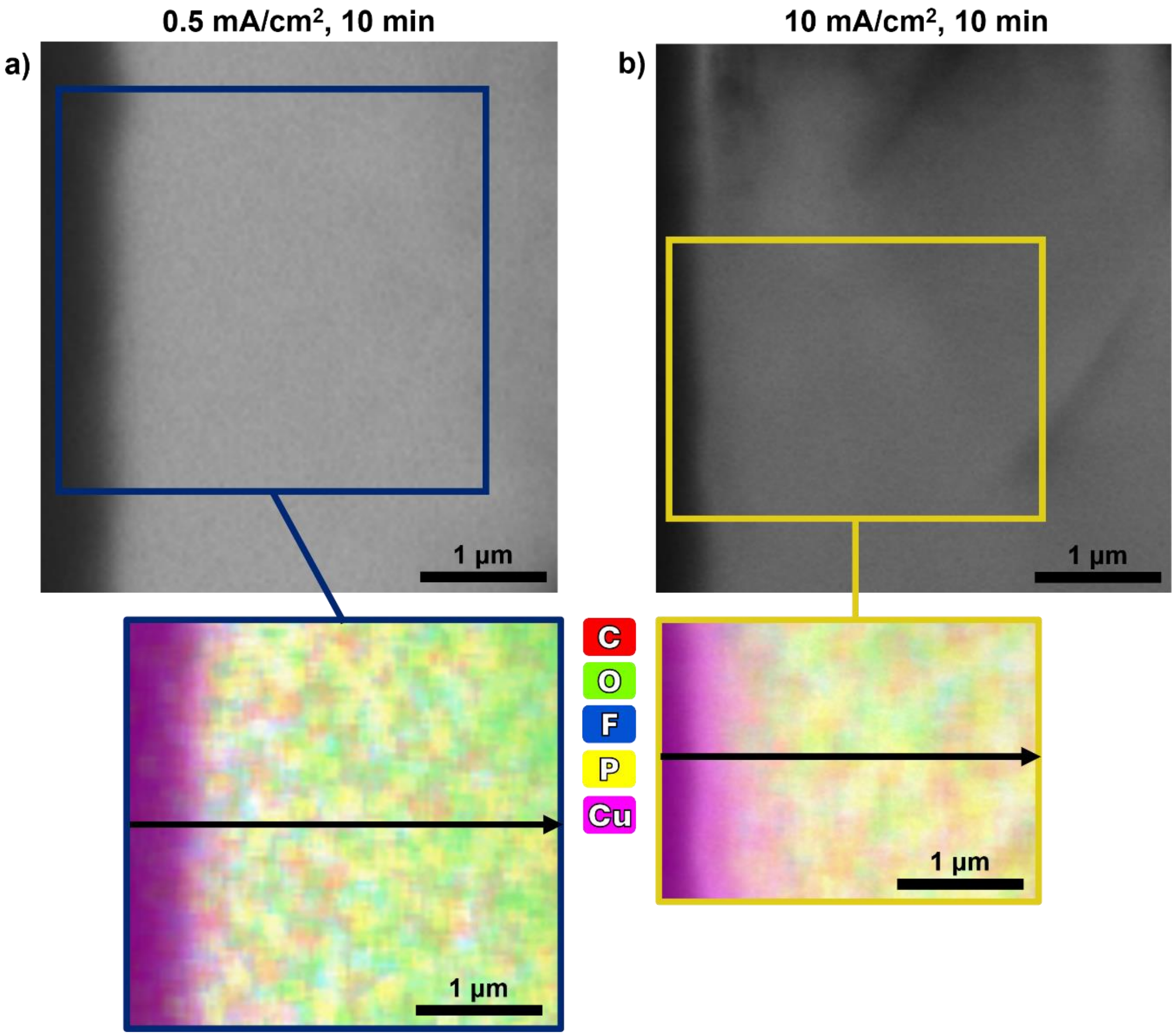

Figure S4: Example cryo-STEM HAADF images used for EDS mapping and corresponding EDS maps and line scans from samples *operando* frozen 10 min into Li deposition at (a) 0.5 mA/cm^2 or (b) 10 mA/cm^2. EDS maps are shown with 10px-average post-processing filter to reduce noise. No structural features besides the edge of the Cu grid bar and uniform layer of vitrified electrolyte are apparent in the 0.5 mA/cm^2 case, consistent with observations of uniform planar Li with minimal growth into pores at this current density. At the higher current density, contrast variation in the electrolyte region is consistent with nonuniform Li growth into the pore, leading to regions with lower Z contrast in HAADF imaging.

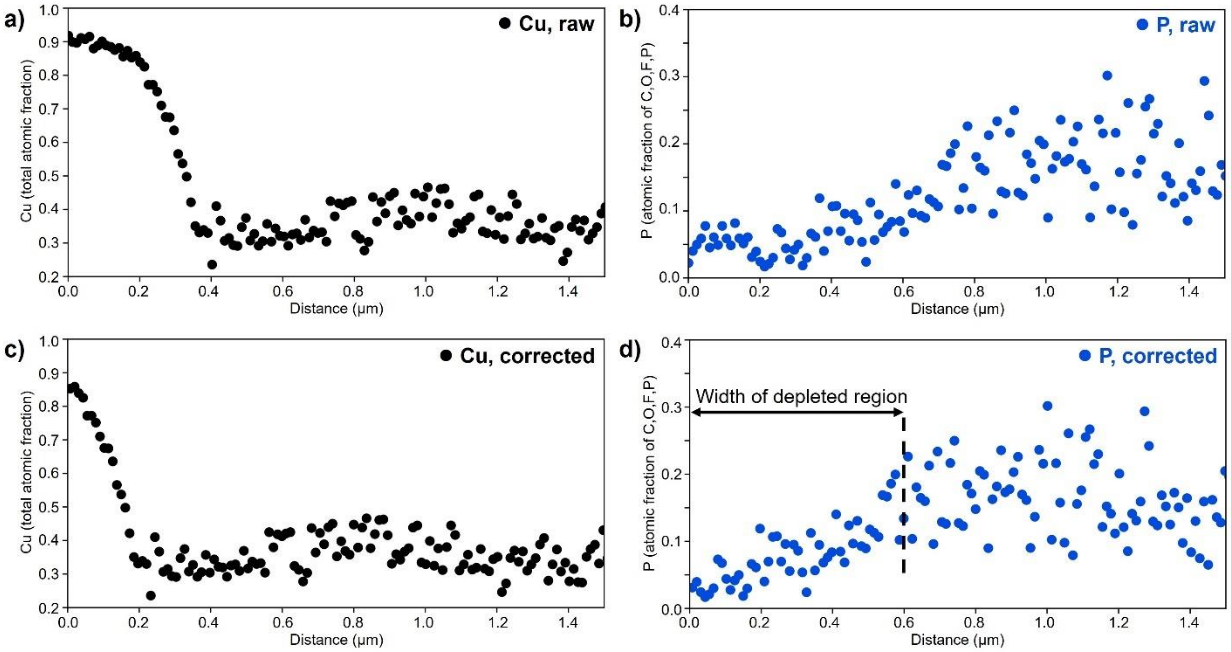

Figure S5: Illustration of calibration of $x = 0$ position in cryo-STEM EDS data, for a sample *operando* frozen at 2 min of Li electrodeposition onto a Cu TEM grid at 10 mA/cm^2. Step 1: Raw EDS data from line scan from Cu grid bar into electrolyte is used to plot (a) the total atomic fraction of Cu (considering all mapped elements) and (b) the P atomic fraction considering only detectable electrolyte elements C, O, F, and P (excluding Cu). The Cu signal shows a steep drop-off, as in (a), corresponding to the end of the grid bar. Step 2: The point of Cu drop-off is used to correct the distance axis of the EDS data, such that $x = 0$ corresponds to the edge of the Cu grid bar for all samples. (c) and (d) show the Cu and P data, respectively, re-plotted after this correction, with the width of the P-depleted region marked by the black arrow and dashed line in (d).

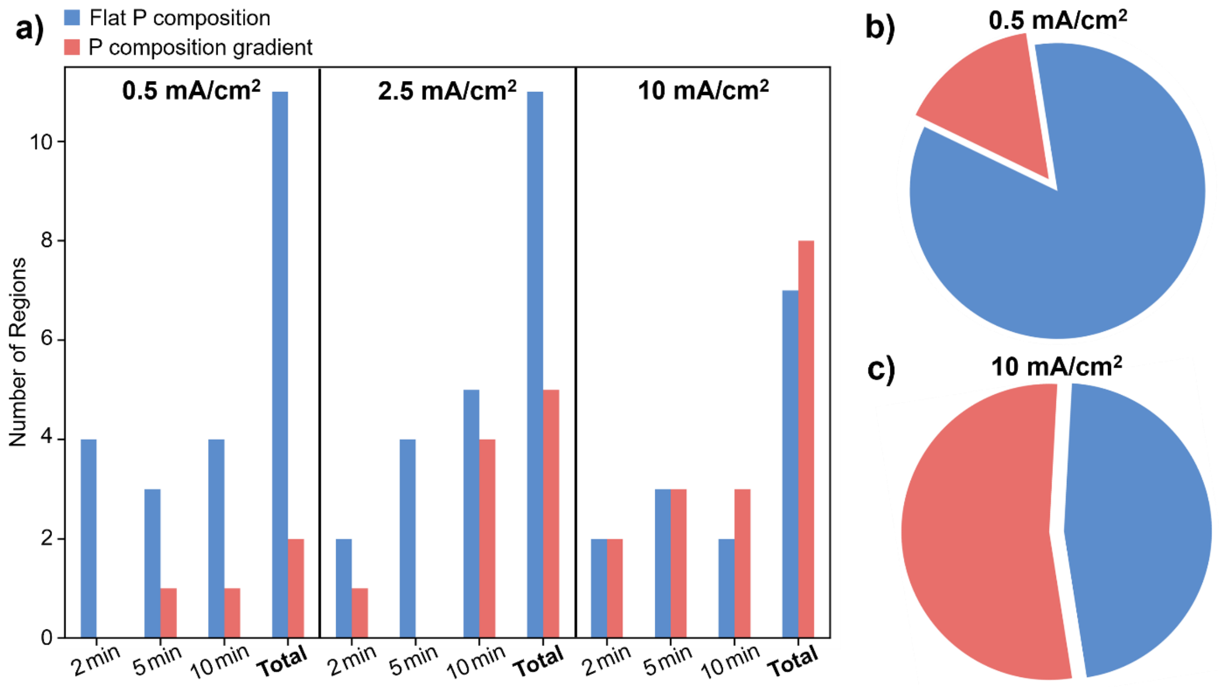

Figure S6: Statistical breakdown of features observed in EDS line scans of *operando* frozen samples. Blue bars and wedges represent regions for which a flat P composition is observed, while red bars and wedges represent regions for which an increasing P composition gradient is observed. (a) Summary of behavior observed for all line scans at 0.5 mA/cm^2, 2.5 mA/cm^2, and 10 mA/cm^2. Regions scanned in samples deposited at 0.5 mA/cm^2 generally show a flat P composition, while a P composition gradient is increasingly prevalent for samples deposited at higher current densities. Pie charts showing all regions scanned for samples deposited at (b) 0.5 mA/cm^2 or (c) 10 mA/cm^2 show a P composition gradient is rare at low current density but observed in the majority of line scans at high current density.

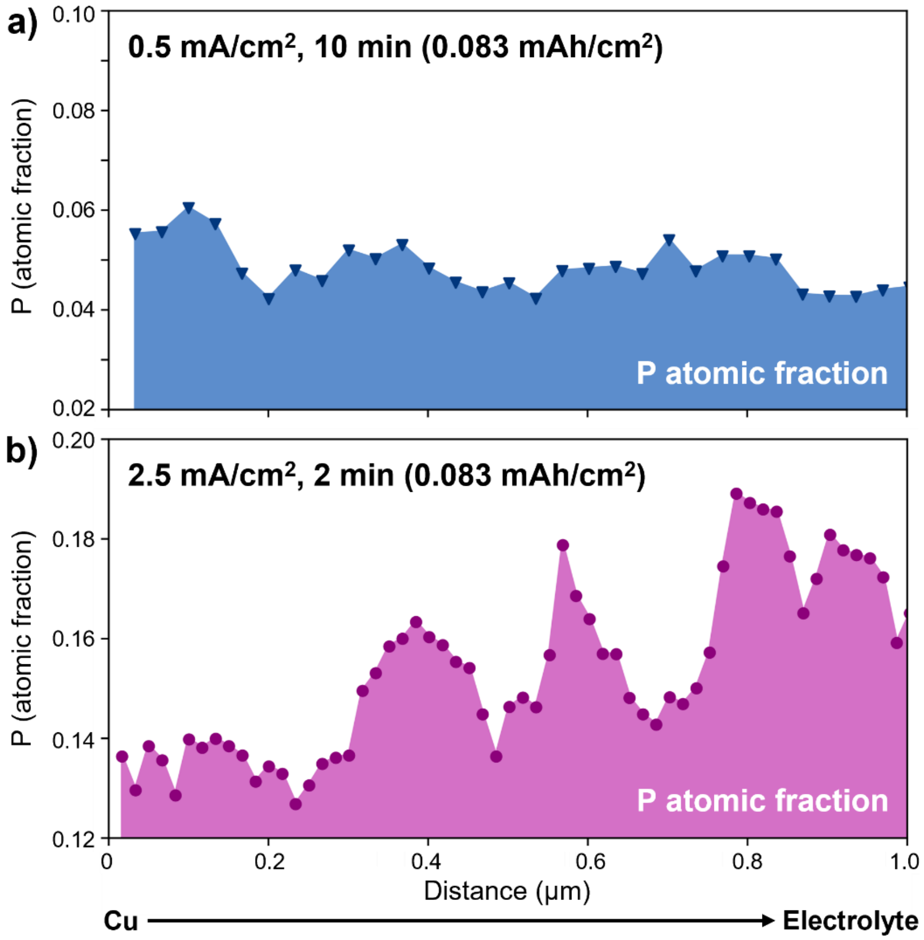

Figure S7: Cryo-STEM EDS phosphorus atomic fraction profiles scanning from Cu current collector to electrolyte for samples *operando* frozen at (a) 10 min of Li electrodeposition at 0.5 mA/cm^2 or (b) 2 min of Li electrodeposition at 2.5 mA/cm^2, both to the same capacity of 0.083 mAh/cm^2, shows the appearance of a P atomic fraction gradient is correlated with current density, rather than capacity.

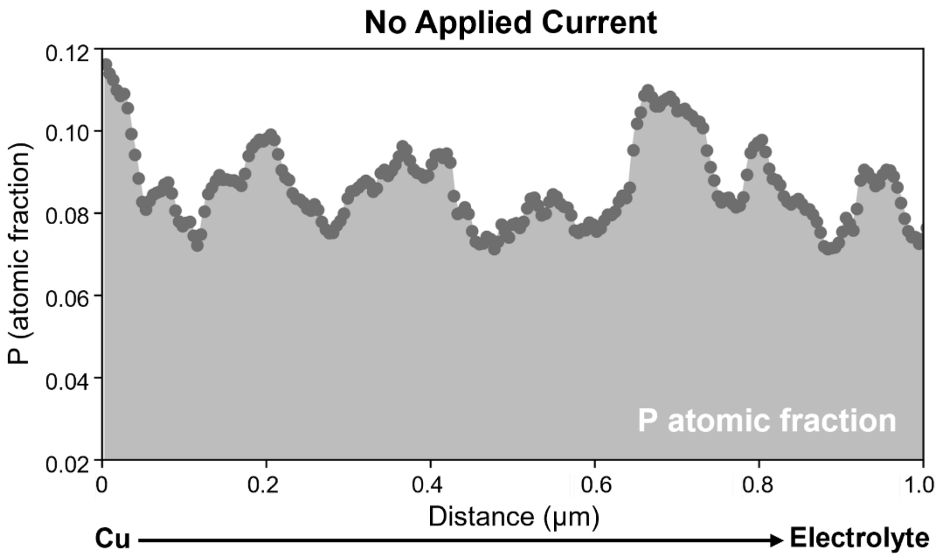

Figure S8: Cryo-STEM EDS phosphorus atomic fraction profile scanning from Cu current collector to electrolyte for sample frozen without any applied current.

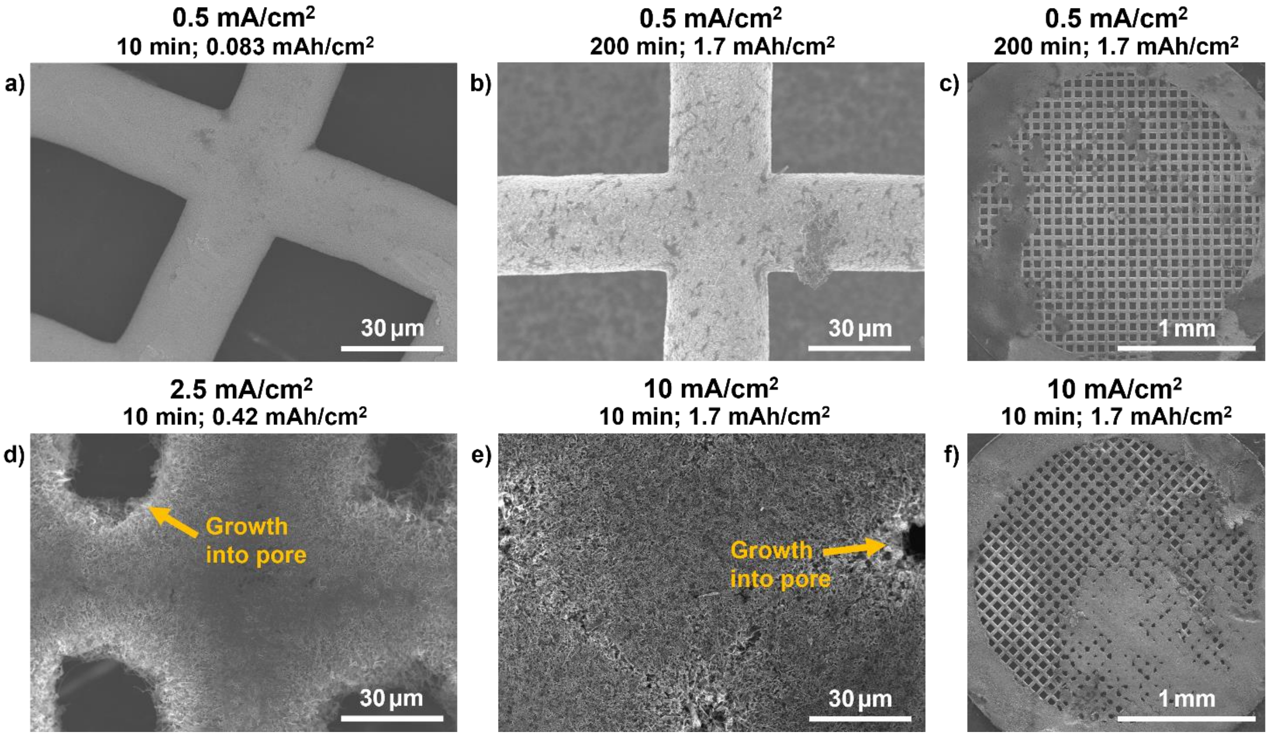

Figure S9: SEM images of Li morphology on Cu grids after (a) 10 min of deposition at 0.5 mA/cm^2 (0.083 mAh/cm^2); (b,c) 200 min of deposition at 0.5 mA/cm^2 (1.7 mAh/cm^2); (d) 10 min of deposition at 2.5 mA/cm^2 (0.42 mAh/cm^2); and (e,f) 10 min of deposition at 10 mA/cm^2 (1.7 mAh/cm^2). Deposition at 0.5 mA/cm^2 generally gives rise to uniform Li grains with a minimal amount of Li moss and negligible growth into the pore of the Cu grid, while 2.5 mA/cm^2 and 10 mA/cm^2 show Li whiskers and extensive growth into the pore. Deposition at 10 mA/cm^2 s is also significantly less uniform across the grid (f) than at 0.5 mA/cm^2 s (c), pointing to the presence of heterogeneous microenvironments.

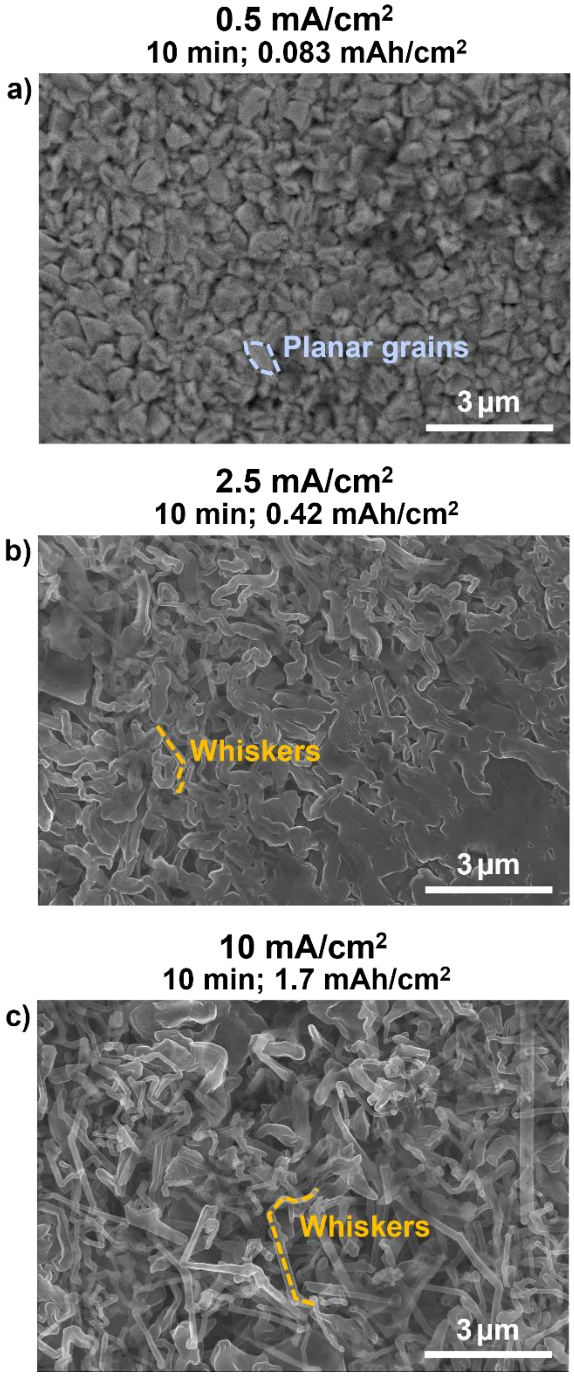

Figure S10: SEM images of Li morphology on Cu grids after 10 min of deposition at (a) 0.5 mA/cm^2 (0.083 mAh/cm^2); (b) 2.5 mA/cm^2 (0.42 mAh/cm^2); and (c) 10 mA/cm^2 (1.7 mAh/cm^2). Deposition at 0.5 mA/cm^2 gives rise to uniform Li grains, while 2.5 mA/cm^2 and 10 mA/cm^2 show Li whiskers.

Finite-Element Model Details

The finite-element model is developed in COMSOL Multiphysics 6.2. The domain includes a uniform Li-ion flux from an "Electrode Surface" with the average current density being specified at the top-most boundary (located at the top of the separator domain in **Fig. S11**), a porous separator domain, and two electrolyte domains with reactive boundaries. In the electrolyte domain, the reactive boundaries are set to the reference potential. **Fig. S12a** illustrates the reactive boundaries. All other boundaries are set to no-flux due to symmetry. Within the electrolyte domain, Li-ion species conservation is resolved using concentrated solution theory of a bipolar salt, which is common in standard pseudo-2D battery models. The governing equation for salt conservation can be expressed mathematically as

$$\frac{\partial(\varepsilon_e c_\mathrm{e})}{\partial t} = \nabla_x \cdot \left(D_e \varepsilon_e^{p_e} \nabla_x c_\mathrm{e} - \mathbf{i}_e \frac{t_+^0}{F} \right),$$

$$\mathbf{i}_e = -\kappa_e \varepsilon_e^{p_e} \nabla_x \Phi_e + 2\,\frac{\kappa_e \varepsilon_e^{p_e} RT}{F}\left(1 + \frac{\partial\, lnf_\pm}{\partial\, c_e}\right)(1 - t_+^0)\nabla_x ln\, c_e\,,$$

Where ε_e is the electrolyte volume fraction, c_e is the salt concentration, D_e is the Li-ion diffusion coefficient, p_e is the Bruggeman coefficient, $\mathbf{i}_e$ is the ionic current in the electrolyte, t_+^0 is the Li-ion transport coefficient, κ_e is the electrolyte ionic conductivity, Φ_e is the electrolyte electrostatic potential, R is the universal gas constant, T is temperature, F is Faraday's constant, and $\left(1 + \frac{\partial\, lnf_\pm}{\partial\, c_e}\right)$ is the thermodynamic factor. The four transport related parameters in the electrolyte are taken for Gen2 electrolytes and can be represented as a function of salt concentration and temperature as

$$\begin{aligned}\kappa_e(c_e, T) = c_e\big(&(0.0001909446\,T^2 - 0.08038545\,T + 9.00341) \\ &+ (-0.00000002887587\,T^4 + 0.00003483638\,T^3 - 0.01583677\,T^2 \\ &+ 3.195295\,T - 241.4638)c_e \\ &+ (0.00000001653786\,T^4 - 0.0000199876\,T^3 + 0.009071155\,T^2 \\ &- 1.828064\,T + 138.0976)c_e^2 \\ &+ (-0.000000002791965\,T^4 + 0.000003377143\,T^3 - 0.001532707\,T^2 \\ &+ 0.3090003\,T - 23.35671)c_e^3\big)\,,\end{aligned}$$

$$\left(1 + \frac{\partial\, lnf_\pm}{\partial\, c_e}\right) = 0.54\, c_e^2\, exp\left(\frac{329}{T}\right) - 0.00225\, c_e exp\left(\frac{1360}{T}\right) + 0.341 exp\left(\frac{261}{T}\right),$$

$$\begin{aligned}&log_{10}\big(1E4\, D_e(c_e, T)\big) \\ &= \Bigg(-0.5688226 - \frac{1607.003}{T - (-24.83763 + 64.07366 c_e)} \\ &+ \left(-0.8108721 + \frac{475.291}{T - (-24.83763 + 64.07366 c_e)}\right) c_e \\ &+ \left(-0.005192312 - \frac{33.43827}{T - (-24.83763 + 64.07366 c_e)}\right) c_e^2\Bigg),\end{aligned}$$

$$\begin{aligned}t_+^0 = &(-0.0000002876102\,T^2 + 0.0002077407\,T - 0.03881203)c_e^2 \\ &+ (0.000001161463\,T^2 - 0.00086825\,T + 0.1777266)c_e \\ &+ (-0.0000006766258\,T^2 + 0.0006389189\,T + 0.3091761).\end{aligned}$$

The “effective” parameters to account for porous media effects (i.e., ε_e, and p_e) are only used in the separator region. In all other regions $\varepsilon_e = 1$. At the Cu reactive boundaries, the Li-ions are removed from the simulation via a Butler—Volmer relationship, which can be expressed as

$$j = \frac{i_0}{F}\left[exp\left(\frac{(1-\alpha)F(\Phi_s - \Phi_e - U_{OCP})}{RT}\right) - exp\left(\frac{-\alpha F(\Phi_s - \Phi_e - U_{OCP})}{RT}\right)\right],$$

where j is the Li-ion flux at the reactive surface, i_0 is the exchange current density, α is the cathodic transference number, Φ_s is the solid-phase potential (set to reference), and U_{OCP} is the open-circuit potential of the Li-plating reaction (set to 0 [V] vs. Li/Li+). **Table S1** documents the model parameters.

Table S1: Constant physics-based model parameters.

Parameter	**Description**	**Value**	**Unit**
$c_{e,0}$	Initial electrolyte concentration	1.2	kmol m^{-3}
T	Temperature	298.15	K
h_{sep}	Length/height of separator	25	µm
$h_{\mathrm{Cu,domain}}$	Length/height of Cu domain	114.3	µm
$h_{\mathrm{test,domain}}$	Length/height of test cell domain	25	µm
$p_{e,sep}$	Bruggeman coefficient of electrolyte in separator	2	-
α	Cathodic transference number	0.5	-
$\epsilon_{el,sep}$	Electrolyte volume fraction in separator	0.4	-
i_0	Exchange current density (set to be fast)	1.3E+2	A m^{-2}
U_{OCP}	Open-circuit potential for Li deposition	0	V
Φ_S	Reactive Cu surface potential (set to reference)	0	V
$d_{Cu,domain}$	Half-length of Cu domain (using symmetry)	76	µm
$d_{sep,domain}$	Half-length of separator domain (using symmetry)	76	µm
$d_{test,domain}$	Half-length of test domain (using symmetry)	27	µm

Importantly, the model described here assumes that the plated Li does not strongly influence the electrolyte volume fraction or overall volume (i.e., deposited Li leaves the model domain). As a back-of-the-hand calculation, if the Li is assumed to plate uniformly on the reactive surface, and that the Li deposits are planar and non-porous, the deposited Li would grow approximately 2 µm into the domain on the depositing surfaces after demanding 10 mA cm^{-2} for 10 min. With a nominal Cu mesh size of 152 µm and a test-cell size of 54 µm, the domain shrinkage due to plated Li is relatively small.

Fig. S12a illustrates the model domain with dimensions described by **Table S1**. **Fig. S12b** illustrates the Li-ion inlet surface (red) and reactive deposition surfaces (blue). Any surface not colored is set to a symmetry (or no-flux) boundary condition. **Fig. S12c-d** illustrate a sample of the model predicted results generated for the 10 mA cm^{-2} condition after 5 min. As shown, the electrolyte current density is uniform at the top of the separator and Li plating reactions occur favorably at the top of the Cu section with progressively less deposition reactions occurring further down the domain. **Fig. S12e-f** indicate how the 3-D salt concentration distribution is averaged/projected in the Test cell (**Fig. S12e**) onto a 2D surface (**Fig. S12f**). Additionally, **Fig. S12f** indicates that the averaged salt concentration provided in the main manuscript is reported along the center symmetry line. **Fig. S13** shows the concentration along this line for 0.5 mA cm^{-2} (**Fig. S13a**) or 10 mA cm^{-2} (**Fig. S13b**) at 2, 5, and 10 min, with y-axes zoomed to show the minor concentration profile predicted, which is negligible in comparison with experimental results.

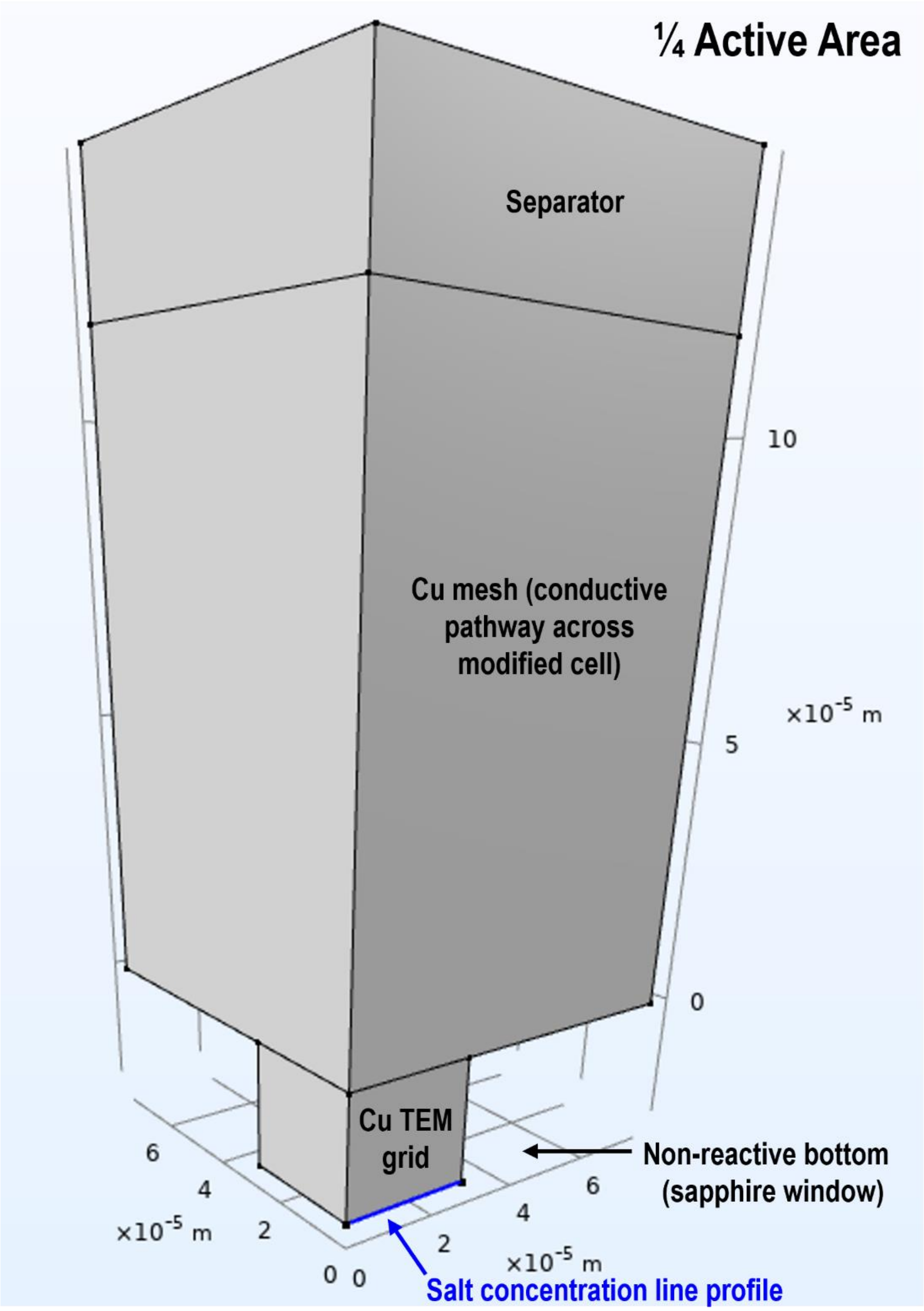

Figure S11: Geometry of finite-element model represents ¼ of the active Li deposition area, reduced by symmetry, and reflects structure of the modified cell, with the Cu TEM grid over a non-reactive surface (cell window), followed by a Cu mesh which provides a conductive pathway across the window, and polymer separator.

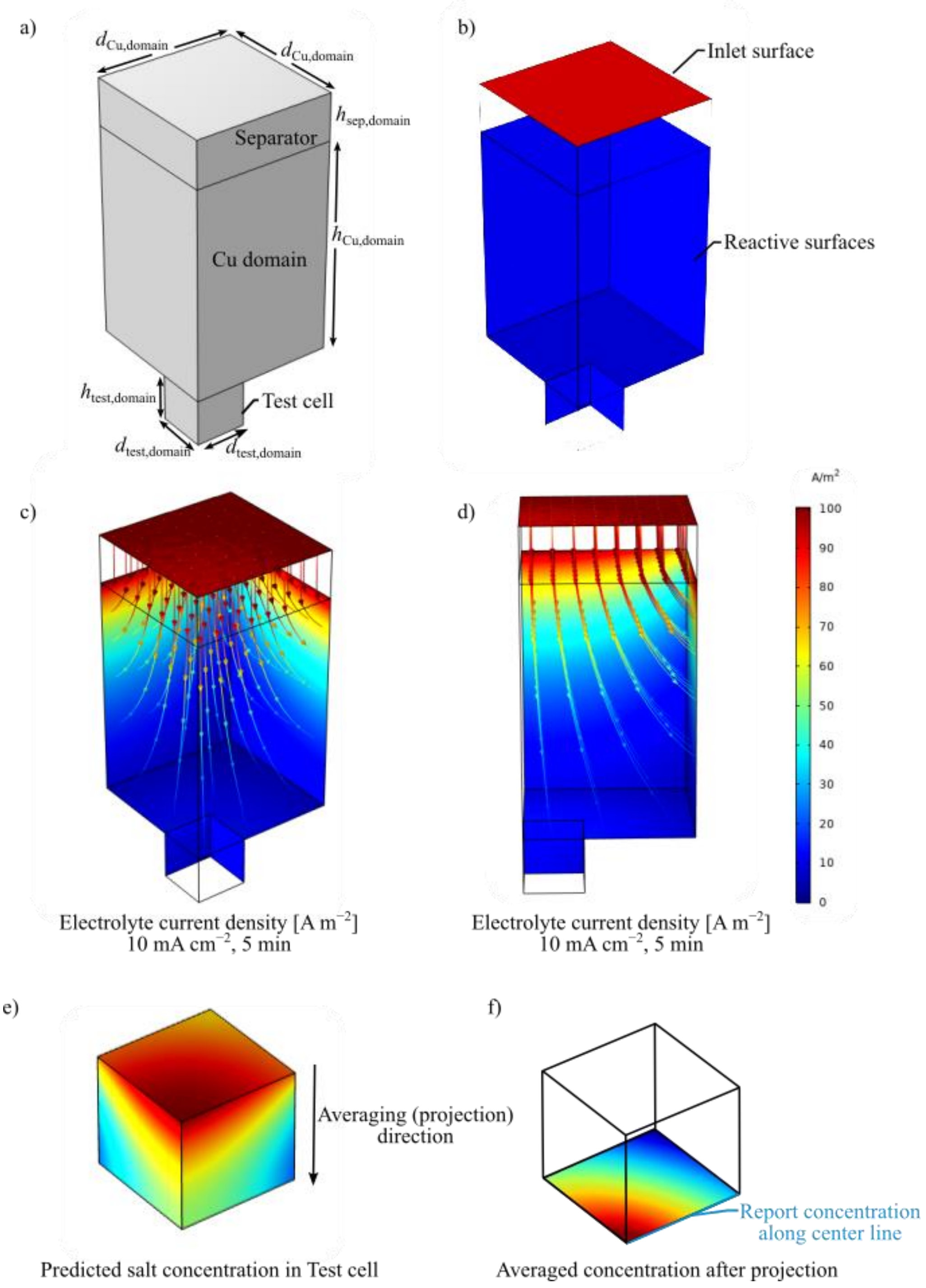

Figure S12: a) Finite-element model domain descripting (representing ¼ of the active Li deposition area), b) inlet and Li-deposition reactive surfaces, c) sample model results of electrolyte current density during the 10 mA cm^{-2} deposition rate after 5 min, d) side-view of electrolyte current density during the 10 mA cm^{-2} deposition after 5 min, e) sample salt concentration predicted by the model in the test cell, f) averaged salt concentration within the test cell projected onto the bottom surface with an indicator for the cell center line where results were extracted.

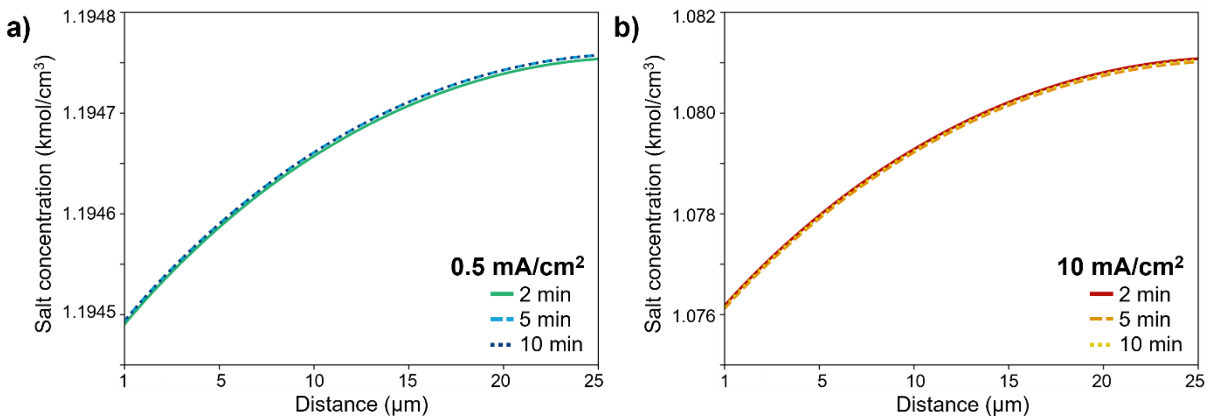

Figure S13: Averaged salt concentration predicted by the finite-element model along the center symmetry line of the Cu pore geometry, for Li deposition at (a) 0.5 mA cm^{-2} or (b) 10 mA cm^{-2}, at 2, 5, or, 10 min. Note the y-axes scales differ between (a) and (b) and are zoomed to show the minor concentration profile predicted by the model, which is negligible in comparison to experimental results.

To compare the modelled salt concentration profile to P atomic fraction profiles measured in EDS, the salt concentration, C, in mol/L of Gen2 electrolyte, was converted to P atomic fraction (considering only EDS-detectable elements in the electrolyte—C, O, P, and F) based on the electrolyte composition using:

$$\text{P atomic fraction} = \frac{C}{7C + 73.1}$$

The modelled and experimental atomic fractions were then normalized by the atomic fraction 1 μm into the electrolyte (for EDS measurements, this was determined by taking an average of the last 5 data points before 1 μm), which represented the "plateau" value for all samples.
